# Longitudinal Analysis of Change in Mammographic Density in Each Breast and Its Association With Breast Cancer Risk

**DOI:** 10.1001/jamaoncol.2023.0434

**Published:** 2023-04-27

**Authors:** Shu Jiang, Debbie L. Bennett, Bernard A. Rosner, Graham A. Colditz

**Affiliations:** 1 Division of Public Health Sciences, Department of Surgery, Washington University School of Medicine in St Louis, St Louis, Missouri; 2 Department of Radiology, Washington University School of Medicine in St Louis, St Louis, Missouri; 3 Channing Division of Network Medicine, Brigham and Women’s Hospital, Boston, Massachusetts

## Abstract

**Importance:**

Although breast density is an established risk factor for breast cancer, longitudinal changes in breast density have not been extensively studied to determine whether this factor is associated with breast cancer risk.

**Objective:**

To prospectively evaluate the association between change in mammographic density in each breast over time and risk of subsequent breast cancer.

**Design, Setting, and Participants:**

This nested case-control cohort study was sampled from the Joanne Knight Breast Health Cohort of 10 481 women free from cancer at entry and observed from November 3, 2008, to October 31, 2020, with routine screening mammograms every 1 to 2 years, providing a measure of breast density. Breast cancer screening was provided for a diverse population of women in the St Louis region. A total of 289 case patients with pathology-confirmed breast cancer were identified, and approximately 2 control participants were sampled for each case according to age at entry and year of enrollment, yielding 658 controls with a total number of 8710 craniocaudal-view mammograms for analysis.

**Exposures:**

Exposures included screening mammograms with volumetric percentage of density, change in volumetric breast density over time, and breast biopsy pathology-confirmed cancer. Breast cancer risk factors were collected via questionnaire at enrollment.

**Main Outcomes and Measures:**

Longitudinal changes over time in each woman’s volumetric breast density by case and control status.

**Results:**

The mean (SD) age of the 947 participants was 56.67 (8.71) years at entry; 141 were Black (14.9%), 763 were White (80.6%), 20 were of other race or ethnicity (2.1%), and 23 did not report this information (2.4%). The mean (SD) interval was 2.0 (1.5) years from last mammogram to date of subsequent breast cancer diagnosis (10th percentile, 1.0 year; 90th percentile, 3.9 years). Breast density decreased over time in both cases and controls. However, there was a significantly slower decrease in rate of decline in density in the breast that developed breast cancer compared with the decline in controls (estimate = 0.027; 95% CI, 0.001-0.053; *P* = .04).

**Conclusions and Relevance:**

This study found that the rate of change in breast density was associated with the risk of subsequent breast cancer. Incorporation of longitudinal changes into existing models could optimize risk stratification and guide more personalized risk management.

## Key Points

**Question:**

Is change in mammographic breast density associated with the development of breast cancer, and does this change diverge from the expected decrease in density with age?

**Findings:**

In this nested case-control cohort study of 947 women attending breast screening during up to 10 years, a decrease in breast density was observed in all women regardless of subsequent breast cancer development. The rate of density change was significantly slower in the breast in which cancer was later diagnosed.

**Meaning:**

This study found that evaluating longitudinal changes in breast density from digital mammograms may offer an additional tool for assessing risk of breast cancer and subsequent risk reduction strategies.

## Teaser

This nested case-control cohort study evaluates the association between change in mammographic density in each breast over time and risk of subsequent breast cancer.

## Introduction

Mammographic breast density is a well-established and strong risk factor for breast cancer.^1,2^ Consistent cross-sectional international data suggest that a decrease in breast density with age is a universal intrinsic biological process in women that is associated with decreasing circulating hormone levels (estrogen and progestin).^3^ Although the assessment of breast density in the radiographic-screening era was qualitative and based on the radiologist’s subjective evaluation of the presence of dense glandular tissue, widespread use of digital mammography created the potential for the quantitative assessment of breast density. The association of breast density with breast cancer risk is consistent across a range of methods used to estimate density as the percentage of density (amount of dense glandular tissue compared with total breast tissue).^4,5^ Volumetric density is an established risk factor for breast cancer and is incorporated into existing risk assessment models, including Tyrer-Cuzick model, version 8.^6^ However, to our knowledge, there are limited data on the association between change in density over time and risk of subsequent breast cancer diagnosis. Retrospective data from Korea show that change in the category of Breast Imaging Reporting and Data System (BI-RADS) breast density is associated with change in risk across 3 mammograms,^7^ consistent with an earlier meta-analysis.^8^ However, this study did not use volumetric analysis of density.

We hypothesized that there is a difference in the rate of change in breast density in women who subsequently develop breast cancer compared with those who do not. Using longitudinal, quantitative data captured for up to 10 years, we evaluated the association between rate of change in mammographic density and risk of breast cancer development.

## Methods

### Study Setting and Ethical Review

Ethical approval for this prospective nested case-control cohort study was obtained from the Washington University in St Louis institutional review board. Participants provided informed written consent. We followed the Strengthening the Reporting of Observational Studies in Epidemiology (STROBE) reporting guideline.

### Description of Cohort

**Figure 1.**
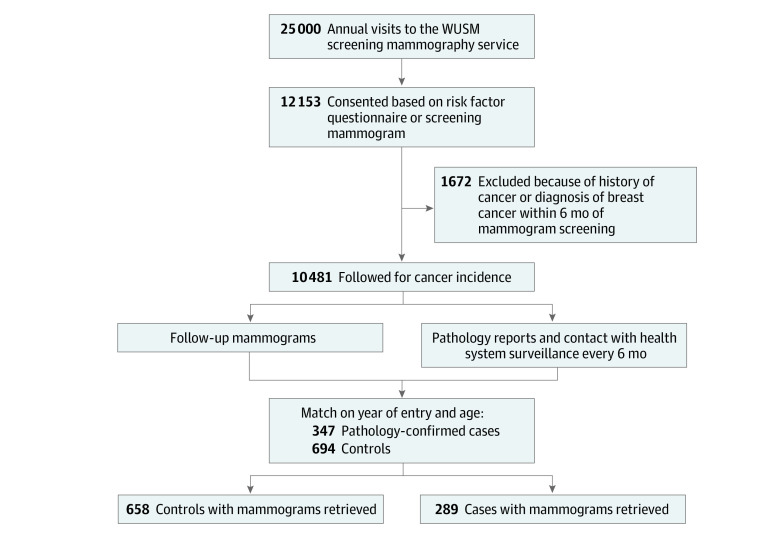
Cohort Recruitment and Follow-up WUSM indicates Washington University School of Medicine.

Women were recruited and provided consent for follow-up from November 3, 2008, to April 30, 2012, through the mammography service at the Joanne Knight Breast Health Center at Washington University in St Louis, Missouri. As previously described, more than 50% of eligible women presenting for screening mammography accepted the invitation to enroll, completed a risk factor questionnaire, and consented to follow-up.^9^ Screening mammograms were obtained from 12 153 women. Of these women, 1672 were excluded for a history of any cancer (except nonmelanoma skin cancer) or a diagnosis of breast cancer within 6 months of registration for the study, for a total of 10 481 women (Figure 1).

Follow-up was conducted by linking to electronic health records, a tumor registry, and a death register. Routine screening mammograms were obtained every 1 to 2 years. A total of 8908 of the 10 481 women in our cohort (85%) had either a mammogram or clinic visit within the last 3 years of follow-up (through October 31, 2020).^9^ The median number of mammograms for each woman in the cohort was 4 (range, 1-10), with an SD of 2.4.

#### Analytic Data Set

We sampled 2 control participants for each case patient according to age at cohort entry and year of enrollment. Incident breast cancers were identified through record linkage to pathology and tumor registries. We identified 347 cases and 694 controls. After linkage to screening mammogram files, we excluded women with breast implants and those without screening mammograms retrieved, and we retained 289 cases and 658 controls with mammograms taken through October 31, 2020, resulting in a total number of 8710 craniocaudal-view mammograms for analysis. All analyses performed in this study used the nested case-control cohort for efficiency of image data processing.

#### Risk Factors Used

Women self-reported breast cancer risk factors on entry to the cohort. These risk factors were drawn from established and validated measures.^10^ The questionnaire at entry assessed height, current weight, parity, age at first birth, cessation of menses (yes or no), age at menopause (natural or with surgical removal of the uterus, with or without removal of the ovaries), age at hysterectomy, family history of breast cancer (mother, sister, or both), history of biopsy-confirmed benign breast disease, current alcohol intake, and race and ethnicity.

#### Volumetric Mammographic Breast Density Assessment

The volumetric density was estimated from each digital craniocaudal-view mammogram with an automated pixel-thresholding algorithm implemented at Washington University on processed images. The skin around the breast is automatically removed with a boundary detection algorithm before estimation of the volume of dense glandular tissue. The volumetric percentage of density is then estimated with the volume of dense glandular tissue divided by the total breast volume; use of the percentage of density normalizes the difference in breast size across women and is consistent with other density estimation methods in the literature.^11,12^ The correlation between volumetric density generated from our automated algorithm with Volpara, version 1.5.0 (Volpara Health) was 0.81 according to an out-of-sample study with 375 women recruited from the mammography service at Washington University with a mean (SD) age of 47 (4.8) years (see eFigure 4 in Supplement 1 for distribution of volumetric density in this cohort).^13,14^

### Analytic Framework

To evaluate hypotheses, we fitted a linear mixed-effects model with separate records for each breast to accommodate longitudinal correlated continuous breast density data.^15^ We fitted a term for breast density in each breast to evaluate differences between cases and controls at entry to the cohort, and then an interaction with time for each breast was used to evaluate the second hypothesis of change in density varying over time (or repeated mammograms with aging) between cases and the controls. With this model, we accounted for correlations within a woman between both her breasts and separately for density measured on the same breast over time. We used Box-Cox transformation to normalize the distribution of breast density and performed model checking and evaluation of residuals (eFigure 1, eFigure 3, and the eAppendix in Supplement 1 for more details). In addition, to replicate standard practice, we also reported a conventional analysis using the mean of density in the 2 breasts.

## Results

Of the 947 women, 141 were Black (14.9%), 763 were White (80.6%), and 20 were of other race or ethnicity (2.1%); 23 patients did not report this information (2.4%). Race and ethnicity were self-reported. The mean (SD) age of the patients was 56.67 (8.71) years at entry. The mean (SD) interval between screening mammograms was 1.3 (0.7) years (10th percentile, 1.0 year; 90th percentile, 2.0 years). For the cases, the mean (SD) time from last mammogram to subsequent breast cancer diagnosis was 2.0 (1.5) years (10th percentile, 1.0 year; 90th percentile, 3.9 years).

The breast cancer risk factors assessed at entry for the women in this study, stratified by case and control status, are presented in Table 1. Most women in both the case and control groups were postmenopausal (209 and 482 women, respectively) and parous (228 and 508 women, respectively). There was no important difference between cases and controls in qualitative breast density assigned by the radiologist at enrollment mammogram (BI-RADS A and B categories [not dense] vs BI-RADS C and D categories [dense]). The correlation of the 2 breasts in the controls at cohort entry was 0.86, and the 2-year correlation within each breast was 0.78 (eFigure 2 in Supplement 1).

**Table 1.** Risk Factors at Mammography by Case-Control Status Within the Prospective Joanne Knight Breast Health Cohort

Factor^a^	No. (%)	
	Cases (n = 289)^b^	Controls (n = 658)
Age, mean (SD), y	56.63 (8.76)	56.67 (8.69)
BMI, mean (SD)	29.27 (6.27)	27.41 (6.18)
No. of longitudinal mammograms, mean (SD)^c^	3.73 (2.18)	4.98 (2.45)
No. of years between mammograms, mean (SD)^c^	1.27 (0.58)	1.35 (0.76)
Time between last mammogram and diagnosis date, mean (SD), y^c^	2.0 (1.5)	NA
BI-RADS category^d^		
A	18 (6.23)	20 (3.04)
B	157 (54.33)	363 (55.17)
C	88 (30.45)	229 (34.80)
D	19 (6.57)	38 (5.78)
Postmenopausal status	209 (72.32)	482 (73.25)
Parous	228 (78.89)	508 (77.20)
Family history of breast cancer	65 (22.49)	125 (19.00)
History of biopsy-confirmed benign breast disease	87 (30.10)	177 (26.90)
Alcohol use	153 (52.94)	411 (62.46)
Race and ethnicity		
Black	56 (19.38)	85 (12.92)
White	228 (78.89)	535 (81.31)
Other	2 (0.69)	18 (2.74)
NR	3 (1.04)	20 (3.04)
Time to cancer, y		
0.5-1	12 (4.15)	NA
>1-2	28 (9.69)	NA
>2-3	36 (12.46)	NA
>3-4	31 (10.73)	NA
>4-5	32 (11.07)	NA
>5-6	43 (14.88)	NA
>6-7	34 (11.76)	NA
>7-8	41 (14.19)	NA
>8-9	24 (8.30)	NA
>9-10	8 (2.77)	NA

Abbreviations: BI-RADS, Breast Imaging Reporting and Data System; BMI, body mass index (calculated as weight in kilograms divided by height in meters squared); NA, not applicable; NR, not reported.

^a^ Continuous covariates are reported as mean (SD); binary covariates are reported by the number of positive responses and their corresponding percentage.

^b^ Case patients who received a diagnosis of breast cancer within the first 6 months since entry were removed.

^c^ Longitudinal mammograms that were obtained within 6 months before diagnosis were removed.

^d^ Categories A and B indicate not dense; C and D, dense.

**Figure 2.**
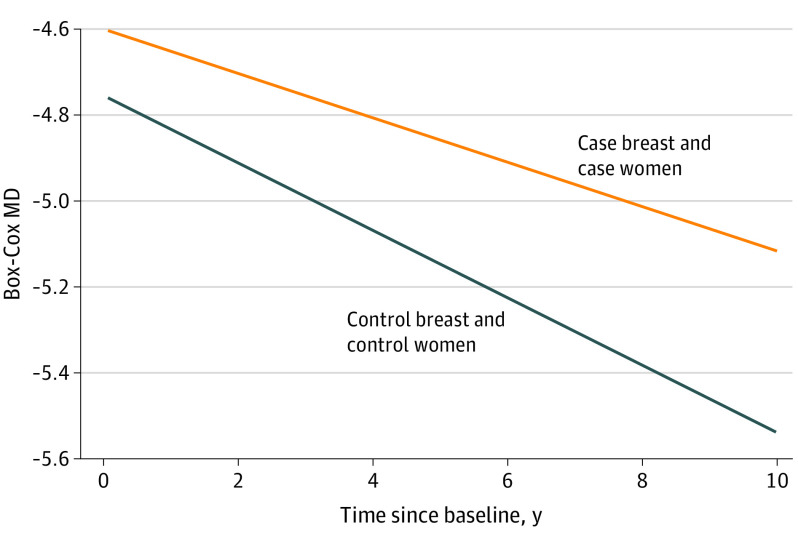
Change in Mammographic Density (MD) for the Case Breast in Case Patients and Control Participants Over Time

When the mean volumetric density of the 2 breasts was used, breast density at entry was significantly higher for cases compared with controls (estimate = 0.140; 95% CI, 0.033-0.246; *P* = .01) (Table 2 and Figure 2). Breast density decreased over time in both groups of postmenopausal women. Women with higher body mass index had lower breast density (estimate = −0.041; 95% CI, −0.049 to −0.033; *P* < .001), and women with a history of biopsy-confirmed benign breast disease had higher breast density (estimate = 0.224; 95% CI, 0.129-0.318; *P* < .001).

**Table 2.** Longitudinal Change in Mean Breast Density Assessed Repeatedly for Up to 10 Years and Association With Breast Cancer^a^

Risk factor^b^	Estimate (95% CI)^c^	*P* value
Age at entry, y	−0.019 (−0.027 to −0.012)	<.001
Follow-up time, y	−0.001 (−0.018 to 0.016)	.92
Case status (case)	0.140 (0.033 to 0.246)	.01
Menopause (post)^d^	0.018 (−0.135 to 0.170)	.82
BMI^d^	−0.041 (−0.049 to −0.033)	<.001
Biopsy-confirmed benign breast disease^d^	0.224 (0.129 to 0.318)	<.001
Family history of breast cancer^d^	−0.006 (−0.110 to 0.098)	.91
Parous (vs nulliparous)^d^	0.071 (−0.030 to 0.171)	.17
Alcohol use (any vs none)^d^	−0.038 (−0.125 to 0.049)	.39
Follow-up time × case status	0.018 (−0.004 to 0.039)	.11
Follow-up time × menopause (post)	−0.058 (−0.077 to −0.039)	<.001
Follow-up time × BMI	−0.006 (−0.007 to −0.005)	<.001

Abbreviation: BMI, body mass index (calculated as weight in kilograms divided by height in meters squared).

^a^ Findings were from 289 women with a diagnosis of breast cancer and 658 control patients from a cohort of 10 481 women (median of 4 mammograms [range, 1-10 mammograms] per woman).

^b^ Age is centered on mean (SD) age of 54 years; BMI, on mean (SD) of 27.

^c^ Box-Cox–transformed volumetric breast density.

^d^ Assessed at entry to study only.

When the mean density of both breasts was used, change in density over time did not differ between cases and controls (represented by follow-up time × case status, estimate = 0.018; 95% CI, −0.004 to 0.039; *P* = .11). However, when density change in each breast was analyzed separately, there was a significant difference in the rate of density change over time in the breast that developed cancer compared with that in controls (estimate = 0.027; 95% CI, 0.001-0.053; *P* = .04) (Table 3). Mammograms for breasts that subsequently developed cancer demonstrated a significantly slower rate of decrease in density than mammograms for breasts that did not later develop cancer in the control women.

**Table 3.** Longitudinal Change Within Each Breast for Breast Density Assessed Repeatedly for Up to 10 Years and Association With Breast Cancer^a^

Risk factor^b^	Estimate (95% CI)^c^	*P* value
Age at entry, y	−0.024 (−0.033 to −0.015)	<.001
Follow-up time, y	−0.001 (−0.017 to 0.015)	.92
Breast did not develop cancer in case patient (vs controls)	0.202 (0.064 to 0.340)	.004
Breast that developed cancer in case patient (vs controls)	0.153 (0.015 to 0.291)	.03
Menopause (post)^d^	0.027 (−0.162 to 0.215)	.78
BMI^d^	−0.051 (−0.060 to −0.042)	<.001
Biopsy-confirmed benign breast disease^d^	0.274 (0.153 to 0.396)	<.001
Family history of breast cancer^d^	−0.018 (−0.151 to 0.116)	.79
Parous (vs nulliparous)^d^	0.087 (−0.042 to 0.217)	.18
Alcohol use (any vs none)^d^	−0.050 (−0.161 to 0.061)	.38
Follow-up time × breast did not develop cancer in case patient (vs controls)	0.020 (−0.006 to 0.046)	.13
Follow-up time × breast that developed cancer in case patient (vs controls)	0.027 (0.001 to 0.053)	.04
Follow-up time × menopause (post)	−0.077 (−0.095 to −0.059)	<.001
Follow-up time × BMI	−0.008 (−0.009 to −0.007)	<.001

Abbreviation: BMI, body mass index (calculated as weight in kilograms divided by height in meters squared).

^a^ Findings were from 289 women with a diagnosis of breast cancer and 658 control patients from a cohort of 10 481 women (median of 4 mammograms [range, 1-10 mammograms] per woman).

^b^ Age is centered on mean (SD) age of 54 years; BMI, on mean (SD) of 27.

^c^ Box-Cox–transformed volumetric breast density.

^d^ Assessed at entry to study only.

To illustrate this significant change in the rate of decrease in density during the follow-up interval, in Figure 2, we show the volumetric density for breasts that subsequently developed cancer (ie, case breast within the cases) compared with the volumetric density for breast mammograms from controls. The decrease in breast density over time was significantly slower for the case breast during the follow-up interval compared with the breasts within the controls. Plots stratified by BI-RADS show similar patterns on the original volumetric density scale and the Box-Cox–transformed scale (eFigure 5 in Supplement 1).

## Discussion

In this prospective cohort study designed to evaluate breast cancer risk over time, we used case-control sampling for efficient processing of 8710 prospectively ascertained digital mammographic images. We observed that breast density was higher at entry to the study for women who would later develop breast cancer compared with controls who remained cancer free. Volumetric breast density decreased significantly over time in both groups. To reflect the underlying biology of breast cancer development, we modeled each breast independently to allow for different rates of change for the breast that would develop breast cancer and the contralateral breast in a case patient that remained free from breast cancer. The rate of decrease in density for the breast that developed breast cancer was significantly slower than for controls who did not develop breast cancer. This observation, using longitudinal repeated measures, reflects the dynamic changes in breast tissue that differ significantly between women who develop breast cancer and those who do not. These data suggest that longitudinal changes in breast density may be used to refine the assessment of risk of breast cancer and to inform precision prevention strategies.

Breast density remains one of the most readily available summary measures from screening mammograms that may be used to assess future breast cancer risk.^16^ Although density is an accepted intermediate marker of breast cancer risk,^2^ studies of change in density are limited. Previous studies have used reader-dependent, subjective measures of density and categorical density data (fatty, scattered, heterogeneously dense, and extremely dense in accordance with BI-RADS).^7,17,18,19,20,21^ Previous studies have also been based on analysis of digitized radiographic images,^7,22,23^ which are of lower image quality than standard digital mammograms.^24,25^ Furthermore, changes in breast density in these studies were often assessed from comparison of only 2 time points. Despite these limitations, a recent meta-analysis of 4 studies reported that a change in BI-RADS breast density category is associated with an increased risk of breast cancer.^8^

Here, we moved beyond these limitations to draw on the full data of digital mammograms to capture changes within each breast over time. Analysis of mammographic images of each breast independently allows for the study of the specific breast in which cancer later develops. An additional strength of this study is the use of a median of 4 (range, 1-10) or more digital mammograms per participant during a follow-up period of 10 years. Follow-up mammograms from 2008 to 2020 reflect current clinical practice. The cohort was generated from a population undergoing routine screening and reflects the broader community.^9^ The mean (SD) time from most recent mammogram to breast cancer diagnosis was 2.0 (1.5) years, and we excluded mammograms within 6 months of diagnosis to avoid bias that can be induced if density is spuriously increased owing to undetected cancer.^4,26^ We used craniocaudal views because epidemiologic studies show that associations of density with breast cancer are stronger for craniocaudal views than for mediolateral oblique views.^27^

Once mammography screening is begun from the age of 45 years (American Cancer Society) or 50 years (US Preventive Services Task Force), repeated either annually or biannually, women accumulate a series of mammograms. Therefore, the available data are naturally composed of longitudinal images for each breast. Traditional models focus on fixed prediction for some future interval conditional on information gathered at baseline. However, given the available longitudinal data, dynamic prediction that can be updated immediately after mammography conditional with the woman’s most up-to-date individualized density trajectory in each breast could improve overall breast cancer risk classification over time. These repeated measures can be further refined for routine practice and incorporated into a dynamic risk classification and hence guide personalized prevention services according to the woman’s level of risk. Further research is needed to define the clinical association between incorporation of density change over time and any subsequent risk reduction strategies that may be recommended as a result.^28,29^ Finally, refining the trade-off of number of images and interval between images to assess the change over time will also inform translation to clinical practice.^30^

In this prospective cohort study using repeated images and established statistical approaches to repeated-measures analysis,^15^ we advanced one-time assessment of breast density, which has already been established as a key component for risk stratification.^31^ A systematic review shows that, in models predicting women’s risk of breast cancer that were published from 2007 to 2019, the addition of a onetime measure of breast density significantly increased discriminatory accuracy in 7 of 11 studies. The increase in the area under the curve ranged from 0.03 to 0.14.^31^ The current findings open the potential for dynamic prediction modeling to account for breast-specific density changes over time.

Much ongoing research focuses on mammography analysis to allow for an earlier diagnosis of breast cancer, when it is the most treatable.^32^ The potential to use the data embedded in mammographic images and make full use of longitudinal measures will facilitate optimizing risk stratification to guide more personalized risk reduction.^33^

### Limitations

The limitations of the study include a population that was predominantly White (763 individuals [80.6%]) and analysis of digital images from only 1 manufacturer (Hologic). However, other studies have shown breast density is a robust risk factor across race and ethnicity and mammography vendors.^3,4,26^

## Conclusions

Using longitudinal digital mammograms of each breast during up to 10 years, this prospective cohort case-control study found that women with a slower decrease in breast density had a higher risk of developing breast cancer. These dynamic changes in density over time may be used to refine risk stratification and guide more individualized screening and prevention approaches.

## Notes

Supplement 1. **eAppendix.** Analytic Framework **eFigure 1.** Top Row Shows Histogram of Mammographic Density of the Two Breasts on the Original Volumetric Scale and the Corresponding QQ Plot for the Normality of Residuals; Bottom Row Shows Mammographic Density on the Box-Cox–Transformed Scale and the Corresponding QQ Plot for the Normality of Residuals With Improved Fit **eFigure 2.** Three Types of Correlations Using Box-Cox–Transformed Breast Densities in the Control Women: *R*_1_ = Correlation Within the Same Breast Over Time; *R*_2_ = Inter-breast Correlation Within the Same Woman; *R*_3_ = Cross-Correlation Between Breasts at Different Time Points **eFigure 3.** Scatter Plot of Box-Cox Transformation vs Original Scale Mammographic Density **eFigure 4.** Volumetric Cut Points for the Original Scale MD to BI-RADS Levels **eFigure 5.** Illustration for Change in MD Stratified by a) BI-RADS A (MD <3.5%) and b) BI-RADS D (MD >15.5%; Bottom Row) for the Case Breast in the Case Women and Control Women Over Time
eReferences.

Supplement 2.
Data Sharing Statement
