# Supplementary material for: Longitudinal Analysis of Change in Mammographic Density in Each Breast and Its Association With Breast Cancer Risk
Source: JAMA Oncol. 2023 Apr 27;9(6):808–14. doi: 10.1001/jamaoncol.2023.0434 (PMC10141289; doi:10.1001/jamaoncol.2023.0434)
Supplement: Supplement 1. — eAppendix. Analytic Framework eFigure 1. Top Row Shows Histogram of Mammographic Density of the Two Breasts on the Original Volumetric Scale and the Corresponding QQ Plot for the Normality of Residuals; Bottom Row Shows Mammographic Density on the Box-Cox–Transformed Scale and the Corresponding QQ Plot for the Normality of Residuals With Improved Fit eFigure 2. Three Types of Correlations Using Box-Cox–Transformed Breast Densities in the Control Women: R1 = Correlation Within the Same Breast Over Time; R2 = Inter-breast Correlation Within the Same Woman; R3 = Cross-Correlation Between Breasts at Different Time Points eFigure 3. Scatter Plot of Box-Cox Transformation vs Original Scale Mammographic Density eFigure 4. Volumetric Cut Points for the Original Scale MD to BI-RADS Levels eFigure 5. Illustration for Change in MD Stratified by a) BI-RADS A (MD <3.5%) and b) BI-RADS D (MD >15.5%; Bottom Row) for the Case Breast in the Case Women and Control Women Over Time eReferences. [file jamaoncol-e230434-s001.pdf]

Supplemental Online Content

Jiang S, Bennett DL, Rosner BA, Colditz GA. Longitudinal analysis of change in mammographic density in each breast and its association with breast cancer risk. *JAMA Oncol*. Published online April 27, 2023. doi:10.1001/jamaoncol.2023.0434

eAppendix. Analytic Framework
eFigure 1. Top Row Shows Histogram of Mammographic Density of the Two Breasts on the Original Volumetric Scale and the Corresponding QQ Plot for the Normality of Residuals; Bottom Row Shows Mammographic Density on the Box-Cox–Transformed Scale and the Corresponding QQ Plot for the Normality of Residuals With Improved Fit
eFigure 2. Three Types of Correlations Using Box-Cox–Transformed Breast Densities in the Control Women: R1 = Correlation Within the Same Breast Over Time; R2 = Inter-breast Correlation Within the Same Woman; R3 = Cross-Correlation Between Breasts at Different Time Points
eFigure 3. Scatter Plot of Box-Cox Transformation vs Original Scale Mammographic Density
eFigure 4. Volumetric Cut Points for the Original Scale MD to BI-RADS Levels
eFigure 5. Illustration for Change in MD Stratified by a) BI-RADS A (MD <3.5%) and b) BI-RADS D (MD >15.5%; Bottom Row) for the Case Breast in the Case Women and Control Women Over Time
eReferences.

This supplemental material has been provided by the authors to give readers additional information about their work.

© 2023 Jiang S et al. *JAMA Oncology.*

eMethods

Analytic framework

Linear mixed effects model with average MD between two breasts. Let Y_{ij} denote the average volumetric mammographic density (MD) between the left and right breast for individual i recorded at time t_{ij}, $i = 1, \dots, n; j = 1, \dots, J_i$. We let $\boldsymbol{X}_i$ denote a length Q vector of baseline risk factors where we considered age, BMI (kg/m^2), biopsy confirmed history of benign breast disease, family history, alcohol, parity, and menopausal status. If we let $I(\delta_i = 1)$ denote the case women who had a breast cancer within the 10 years of follow-up in the cohort, we can construct the linear mixed effects model as,

$$\mathrm{Y_{ij}} = \beta_0 + \beta_1 I(\delta_i = 1) + \beta_2 t_{ij} + \beta_3 I(\delta_i = 1) t_{ij} + \alpha_1 X_{i1} + \cdots + \alpha_Q X_{iQ} + \gamma_1 X_{i1} t_{ij} + \cdots + \gamma_R X_{iR} t_{ij} + u_i + e_{ij}, \quad (1)$$

where we assume that we have Q baseline risk factors and R interactions with time, $R \leq Q$. The average MD was transformed using the Box-Cox transformation to satisfy the normality assumption in the mixed effects model in accordance with the literature.[1] The Box-Cox transformation is defined as a function a power parameter λ, i.e., $Y = (Y^{\lambda} - 1)/\lambda$ if $\lambda \neq 0$, and $Y = \log{(Y)}$ if $\lambda = 0$. The estimation for the power parameter λ can be carried out with the *R* function *boxcox*. The estimated λ for our data using average MD between two breasts is -0.18.

The time-invariant parameters are as follows: β_0 is the intercept that denotes the population average density over all time points, β_1 is the comparison of density for case women vs. control women [$I(\delta_i = 0)$] at baseline, $\alpha_1 \dots \alpha_Q$ is the vector of coefficients of the baseline risk factors, u_i is the random intercept for the ith woman, and e_{ij} is the residual error. We assume that $u_i \sim N(0, \sigma_u^2)$ and e_{ij} are i.i.d. with mean 0 and variance σ^2. It is assumed that u_i and e_{ij} are

© 2023 Jiang S et al. *JAMA Oncology.*

mutually independent. Fixed effects are constant across women, whereas random effects vary across women. In our analysis, a random intercept for MD was fitted such that women starting or entering the cohort at different levels of breast density is accommodated. Other variables are considered as fixed effects.

The time-related parameters, on the other hand, are defined as follows: β_2 is the slope that denotes the change of MD over time in control women, i.e., the population level, and β_3 is the change in MD over time for case women. Here, we note that testing the following set of hypotheses,

$$H_0: \beta_2 = 0 \quad vs. \quad H_1: \beta_2 \neq 0 \qquad (2)$$

enables us to assess whether the change of MD over time is significant in the population. On the other hand, testing the following,

$$H_0: \beta_3 = 0 \quad vs. \quad H_1: \beta_3 \neq 0 \qquad (3)$$

enables us to assess whether the change of MD over time is significantly different between the cases and controls in the cohort.

Linear mixed effects model with MD in each breast. Instead of averaging MD between the two breasts, we can further investigate whether the longitudinal profile of the breast that goes on to develop breast cancer is different from the profile of the breast that does not, and from women who do not develop breast cancer during follow up.[2] We let Y_{ijk} denote the Box-Cox transformed MD of the ith woman, taken at time t_{ij}, for the kth breast, $k = 1,2$. The estimated λ for our data using MD in each breast is -0.26. We then construct an indicator variable with three levels that corresponds to:

$$D_{ik}^{(1)} = I(i\text{th woman is a control}, k\text{th breast is a breast without breast cancer}); \qquad (4)$$

© 2023 Jiang S et al. *JAMA Oncology.*

$D_{ik}^{(2)} = I(i\text{th}$ woman is a case, $k\text{th}$ breast is a breast without breast cancer $)$;

$D_{ik}^{(3)} = I(i\text{th}$ woman is a case, $k\text{th}$ breast is a breast that develops breast cancer $)$.

For a particular women i, breast k, only one of these indicators can take on a value of 1, and the rest 0. As these indicators are assumed to be the observed event by the end of the follow up, they do not have an indicator j. Similar to equation (1), we consider a random-intercept mixed effects model using $D_{ik}^{(1)}$ as the reference level:

$$\mathrm{Y_{ijk}} = \beta_0 + \beta_1 t_{ij} + \beta_2 D_{ik}^{(2)} + \beta_3 D_{ik}^{(3)} + \beta_4 D_{ik}^{(2)} t_{ij} + \beta_5 D_{ik}^{(3)} t_{ij} + \alpha_1 X_{i1} + \cdots + \alpha_Q X_{iQ} + \gamma_1 X_{i1} t_{ij} + \cdots + \gamma_R X_{iR} t_{ij} + u_i + e_{ij}, \quad (5)$$

where we have accounted for three types of correlations: (i) the cross-sectional inter-breast correlation; (ii) the longitudinal correlation among repeated measures in the same breast over time; and (iii) the cross-correlation between the MD of one breast at one time point and the MD for the contralateral breast at a different time point.

As such, testing the following set of hypotheses,

$$H_0: \beta_2 = 0 \quad vs. \quad H_1: \beta_2 \neq 0 \quad (6a)$$

enables us to assess whether the breast without breast cancer within the case women is different from the control women at baseline. On the other hand, testing the following,

$$H_0: \beta_4 = 0 \quad vs. \quad H_1: \beta_4 \neq 0 \quad (6b)$$

enables us to assess whether the change for breast without breast cancer within the case women is different from the control women.

Further, testing the following set of hypotheses,

$$H_0: \beta_3 = 0 \quad vs. \quad H_1: \beta_3 \neq 0 \quad (7a)$$

enables us to assess whether the breast that develops breast cancer within the case women is different from the control women at baseline. Similarly, testing for

© 2023 Jiang S et al. *JAMA Oncology.*

$$H_0: \beta_5 = 0 \quad vs. \quad H_1: \beta_5 \neq 0 \tag{7b}$$

enables us to assess whether the change for breast that develops breast cancer within the case women is different from the control women.

Estimation. We fit both linear mixed effects models using existing *R* package *lmer.*

Model checking. Justification and illustration for using the Box-Cox in comparison to the square-root transformation are demonstrated in eFigure 1 within the Supplementary Material. The normality of the data is further improved with the Box-Cox transformation, moving away from the right skewed MD distribution. A scatter plot of Box-Cox transformation vs. MD is also illustrated in eFigure 3. Further, we performed tests of assumptions for all linear mixed effects models used in this paper. This includes testing for the normality of residuals. See eFigures 1 for these plots. All results that use the term 'MD' in the following subsections refer to the Box-Cox transformed MD.

eResults

To aid interpretation in evaluating change in MD over time in each breast, we give an example here. For a 54 year old postmenopausal women with mean BMI and no risk factors, the decrease in MD in either breasts per year for the control women free from cancer is -0.077 per year. Within women that will develop breast cancer during follow-up, the decrease in MD for the breast that is free from cancer is slower, that is -0.077 + 0.020 = -0.057 per year, and for the breast that will develop breast cancer 0.077 +0.027 = -0.050 per year. Thus, the MD of the breasts will significantly diverge over time between the case breast and control women. (Figure 2 in the main manuscript)

© 2023 Jiang S et al. *JAMA Oncology.*

Figures

eFigure 1. Top row shows histogram of MD of the two breasts on the original volumetric scale and the corresponding QQ plot for the normality of residuals; Bottom row shows MD on the Box-Cox transformed scale and the corresponding QQ plot for the normality of residuals with improved fit.

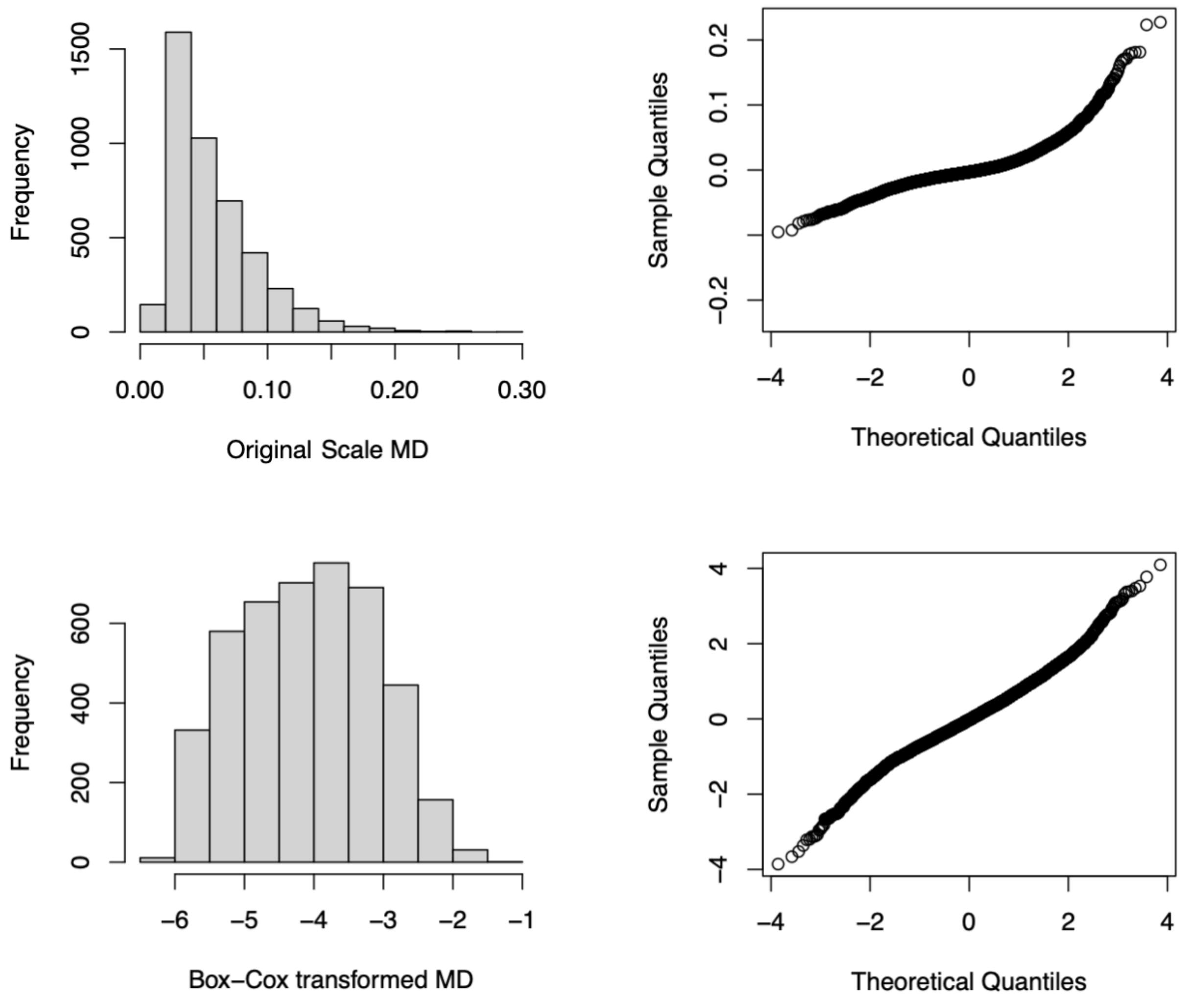

© 2023 Jiang S et al. *JAMA Oncology.*

eFigure 2. Three types of correlations using Box-Cox transformed breast densities in the control women: R_1 = correlation within the same breast over time; R_2 = inter-breast correlation within the same woman; R_3 = cross-correlation between breasts at different time points.

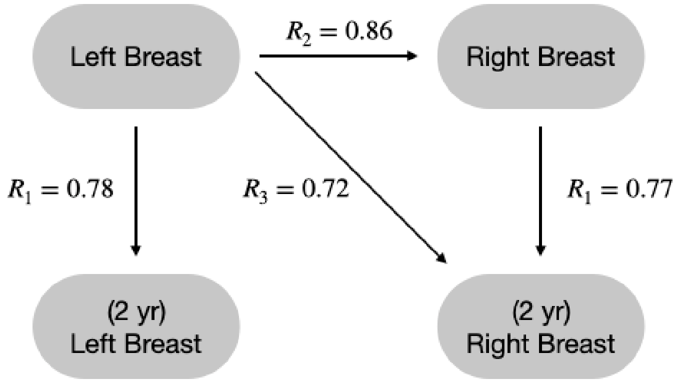

eFigure 3. Scatter plot of Box-Cox transformation vs. original scale MD.

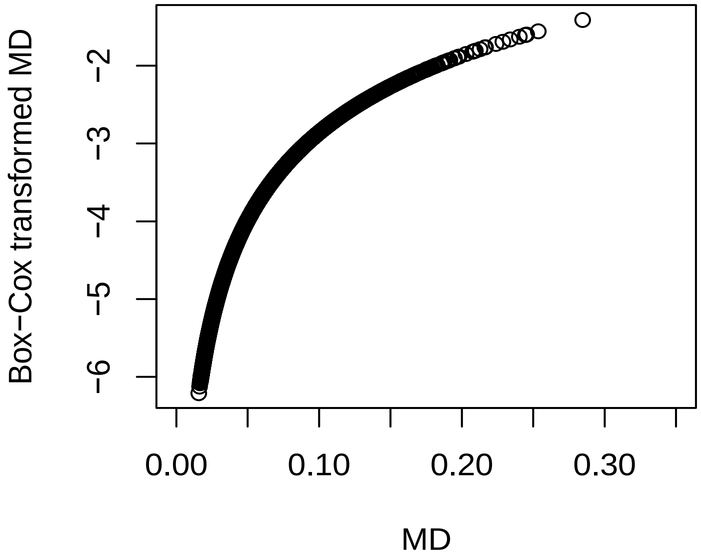

© 2023 Jiang S et al. *JAMA Oncology.*

eFigure 4. Volumetric cut points for the original scale MD to BI-RADS levels. Corresponding to the Breast Imaging Reporting and Data System categorical terms (5th edition), these percentages translate to (a) <3.5%; (b) ≥3.5 and <7.5%; (c) ≥7.5 and <15.5%; and (d) ≥15.5%[1]

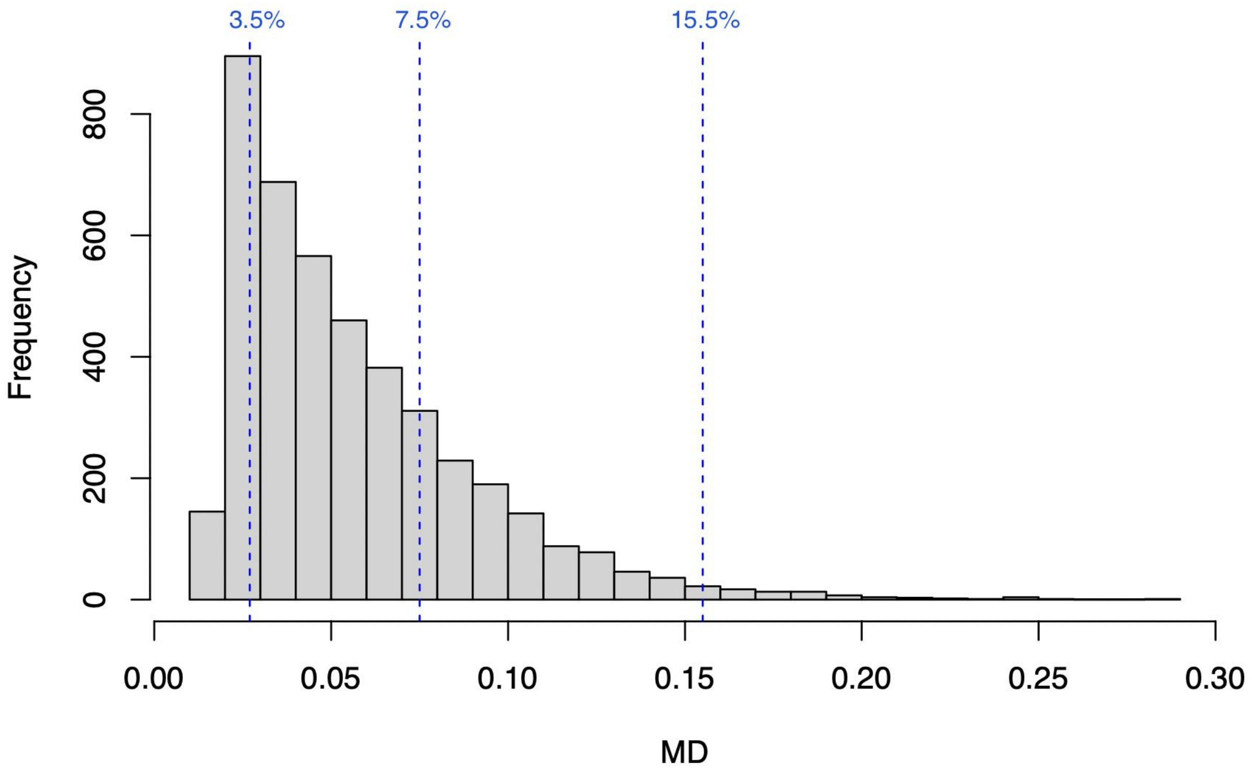

© 2023 Jiang S et al. *JAMA Oncology.*

eFigure 5. Illustration for change in MD stratified by a) BI-RADS A (MD < 3.5%) and b) BI-RADS D (MD > 15.5%; bottom row) for the case breast in the case women and control women over time. The change on the original volumetric percent scale is shown in the first column. The Box-Cox transformed change over time is shown on the second column.

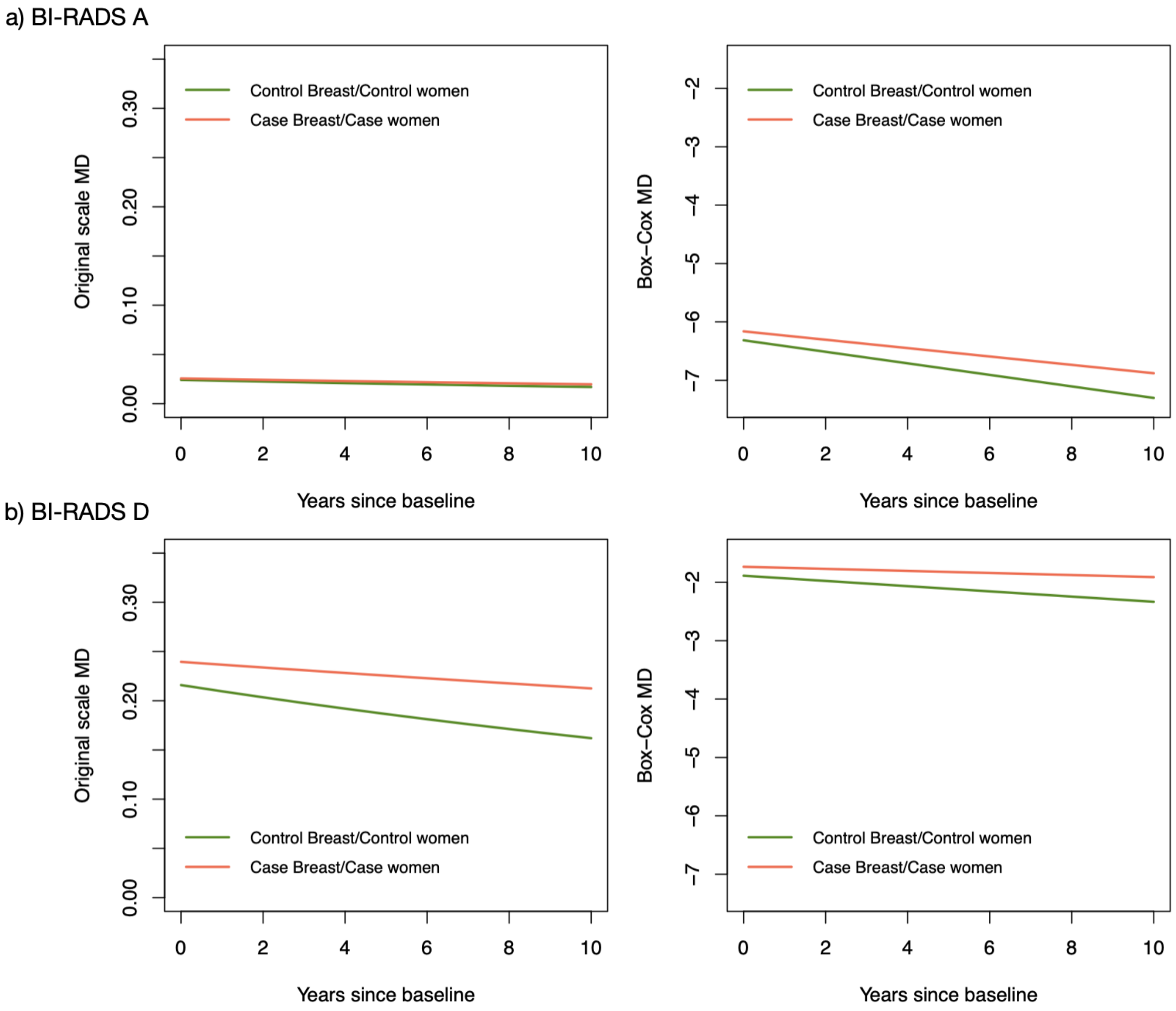

For women who are postmenopausal with average BMI and age and no other risk factors.

1. Technology VSfM. *Volpara DensityTM user manual version 1.5.0.* 2013.

© 2023 Jiang S et al. *JAMA Oncology.*

References

1. Nguyen TL, Choi YH, Aung YK, et al. Breast Cancer Risk Associations with Digital Mammographic Density by Pixel Brightness Threshold and Mammographic System. *Radiology*. Feb 2018;286(2):433-442. doi:10.1148/radiol.2017170306
2. Ying G-S, Maguire MG, Glynn RJ, Rosner B. Tutorial on Biostatistics: Longitudinal Analysis of Correlated Continuous Eye Data. *Ophthalmic Epidemiology*. 2020:1-18.

© 2023 Jiang S et al. *JAMA Oncology.*
