# Supplementary material for: Longitudinal Analysis of Change in Mammographic Density in Each Breast and Its Association With Breast Cancer Risk
Source: JAMA Oncol. 2023 Apr 27;9(6):808–14. doi: 10.1001/jamaoncol.2023.0434 (PMC10141289; doi:10.1001/jamaoncol.2023.0434)
Supplement: Supplement 2. — Data Sharing Statement [file jamaoncol-e230434-s002.pdf]

Data Sharing Statement

Jiang. Longitudinal Analysis of Change in Mammographic Density in Each Breast and Its Association With Breast Cancer Risk. *JAMA Oncol*. Published April 27, 2023. doi:10.1001/jamaoncol.2023.0434

Data
Data available: Yes
Data types: Deidentified participant data, Data dictionary
How to access data: not ready yet but likely through university library system
When available: With publication

Supporting Documents
Document types: None

Additional Information
Who can access the data: anyone requesting data
Types of analyses: for any purpose
Mechanisms of data availability: through library data sharing system being implemented by WashU
